# Respiratory Prediction Based on Multi-Scale Temporal Convolutional Network for Tracking Thoracic Tumor Movement

**DOI:** 10.3389/fonc.2022.884523

**Published:** 2022-05-27

**Authors:** Lijuan Shi, Shuai Han, Jian Zhao, Zhejun Kuang, Weipeng Jing, Yuqing Cui, Zhanpeng Zhu

**Affiliations:** ^1^College of Electronic Information Engineering, Changchun University, Changchun, China; ^2^Jilin Provincial Key Laboratory of Human Health Status Identification and Function Enhancement, Changchun, China; ^3^College of Computer Science and Technology, Changchun University, Changchun, China; ^4^College of Information and Computer Engineering, Northeast Forestry University, Harbin, China; ^5^Department of Neurosurgery, The First Hospital of Jilin University, Changchun, China

**Keywords:** radiotherapy, respiratory motion prediction, deep learning network, empirical mode decomposition, temporal convolutional network

## Abstract

Radiotherapy is one of the important treatments for malignant tumors. The precision of radiotherapy is affected by the respiratory motion of human body, so real-time motion tracking for thoracoabdominal tumors is of great significance to improve the efficacy of radiotherapy. This paper aims to establish a highly precise and efficient prediction model, thus proposing to apply a depth prediction model composed of multi-scale enhanced convolution neural network and temporal convolutional network based on empirical mode decomposition (EMD) in respiratory prediction with different delay times. First, to enhance the precision, the unstable original sequence is decomposed into several intrinsic mode functions (IMFs) by EMD, and then, a depth prediction model of parallel enhanced convolution structure and temporal convolutional network with the characteristics specific to IMFs is built, and finally training on the respiratory motion dataset of 103 patients with malignant tumors is conducted. The prediction precision and time efficiency of the model are compared at different levels with those of the other three depth prediction models so as to evaluate the performance of the model. The result shows that the respiratory motion prediction model determined in this paper has superior prediction performance under different lengths of input data and delay time, and, furthermore, the network update time is shortened by about 60%. The method proposed in this paper will greatly improve the precision of radiotherapy and shorten the radiotherapy time, which is of great application value.

## 1 Introduction

When a patient with cancer undergoes radiation therapy, the fluctuating movement of chest and abdomen caused by human respiratory motion makes the tumor unable to rest statically in the planning target volume (PTV), which causes it impossible to ensure the coverage of tumor by simply increasing the PTV area. Meanwhile, it is very likely for the organs at risk (OARs) around the tumor to be destroyed during radiotherapy, thus causing secondary injury to the patients (1). Some studies have shown that, during breathing, some muscles (such as the diaphragm) move 20–130 mm, the lungs move an average of 8–10 mm, and the liver moves an average of 1–19 mm (2). Therefore, it is of great significance to reduce the adverse effects of human respiratory movement in the process of cancer treatment.

To address the problem of respiration-induced tumor displacement, many clinical initiatives have been proposed, including breath-holding techniques (3), passive compression techniques (4), respiratory gating techniques (5), and real-time tracking techniques (6). Breath-holding technique and passive compression technique both reduce the impact by actively controlling human respiration by itself or external equipment, which is very convenient, but the mandatory control makes the patient’s tolerance poor and is not suitable for patients with pulmonary insufficiency. Respiratory gating technology tracks the location of the tumor by monitoring the patient’s breathing and adjusting the radiation instrument to match a specific breathing cycle. Real-time tracking technology is currently one of the best methods to track tumors and improve treatment effects. It continuously adjusts the irradiation target area to track tumors in real time through *in vitro* marker signals (respiration laws).

Vedam et al. (7), Ozhasoglu and Murphy (8), and Fayad et al. (9) verified the correlation between respiration and tumor movement to varying degrees. CyberKnife, Exactrac, and Vero system are respiratory motion tracking systems applied in clinical practice. In the actual treatment, the machine system establishes the motion relationship between marker signals and tumor through the prediction model, so as to adjust the radiotherapy target position. A certain time delay is required during the adjustment process, which demands the establishment of prediction delay system through the external respiratory signal. The accuracy of delay prediction directly determines the target position in radiotherapy. The CyberKnife system has a system delay time of about 115 ms from data acquisition, calculation of tumor location, to adjustment of the radiation beam. The delay of Vero system is about 50 ms and that of Varian MLC system is about 420 ms (10, 11). To compensate for these delays, some prediction algorithm is used to calculate the future position of the target.

Conventional time series prediction models have been applied in the field of respiratory prediction, such as extended Kalman filter algorithm based on Kalman filter (12) combining with support vector machine (13), wavelet-based multi-scale regression (14), recursive least squares algorithm (15), and an autoregressive integrated moving average (ARIMA) model (16). With the development of deep learning, it has brought new possibilities to respiratory motion prediction. Deep learning can effectively mine time series information and semantic information, independently extract a large number of data features, and improve the prediction accuracy. To compensate for the system delay and improve the accuracy of respiratory motion prediction, this paper proposes a multi-scale enhanced time series convolution respiratory motion prediction model based on deep learning network. The main contributions are as follows:

(1) A multi-scale enhanced convolution and temporal convolution network (TCN) based on squeeze-and-excitation is proposed to establish a deep convolution neural network model for respiratory motion prediction.
(2) Aiming at the simplification of respiratory signal features, EMD algorithm is used to decompose the original complex sequence into several intrinsic mode functions (IMFs) with different time scales so as to increase the network fitting ability and improve the prediction precision.
(3) The underlying features of different receptive fields are extracted by using a multi-scale convolution kernel, and attention mechanisms are added to the feature space.
(4) The recurrent neural network (RNN) model is replaced by the TCN, which has higher precision and time efficiency than birectional long short-term memory (BiLSTM).

## 2 Related Work

Deep learning is based on artificial neural network (ANN), which has stronger adaptability in the case of irregular breathing model and model. Some studies have shown (17, 18) that the ANN structure has certain advantages in the prediction of respiratory motion, especially when the respiratory signal is unstable and non-linear.

Convolutional neural network (CNN) can deal with data similar to grid structure through convolution operation and perform exceedingly well in many fields such as time series and image data; RNN has some advantages when learning the non-linear characteristics of sequences. LSTM is one of the classical algorithms of RNN series because of its introduction of the gate mechanism to make the network have a certain memory, so that the network can capture the long-distance dependence of the sequence and better overcome the disadvantage of gradient disappearance in RNN. This deep learning mechanism allows the automatic construction of a model from a problem or set of rules. When dealing with large amounts of data, the model can adapt to input new data or import new knowledge through other models, allowing it to solve almost any real-world task (19). Wang et al. (20) established BiLSTM network by composing forward and backward LSTM and applied it in the experiment respiratory data of 103 patients with malignant tumors. Through the experiment, they found that the best prediction effect was obtained when seven-slice BiLSTM was used, with an average absolute error of 0.074 mm and a root mean square error (RMSE) of 0.097 mm at a delay standard of 400 ms, which was three to five times higher than the prediction precision of ARIMA and multi-layer perceptron neural network (ADMLP-NN). Compared with traditional prediction models, the deep learning network with higher robustness can greatly improve the prediction precision, which can be applied to data of different patients and reduce the interference of delay time. However, deeper network will lead to longer update time of prediction, which is not conducive to the update of prediction model. The Bidirectional Gated Recurrent Unit (Bi-GRU) rapid breathing prediction model was constructed by Yu et al. (21) by using a variant of LSTM–gating cycle unit (GRU), consequently reducing the time efficiency by about 30% compared with the LSTM model, which greatly improved the update time of the prediction network. Therefore, deep learning will be an emerging force driving progress in the field of respiratory motion prediction.

In general, the prediction accuracy of the model can be greatly improved by training the model on the clinical data of a limited number of patients (18, 22). However, when the model is applied to new patient data, the prediction effect is greatly discounted, and the generalization ability of the prediction model needs to be improved. Each patient has different physical conditions and respiratory states, and it is of great significance to design a general model to predict the respiratory signals of different patients (23). The establishment of a general model requires a large amount of patient data as support, so deep learning has good applicability, because deep learning has better learning and analysis capabilities under a large amount of data.

## 3 Materials And Methods

### 3.1 Respiratory Movement Data

The data used in this paper are a publicly available dataset derived from the Institute of Robotics and Cognitive Systems, University of Lubeck, Germany (24). This dataset contains the respiratory data of 103 patients with horacoabdominal tumors, with a total of 306 respiratory motion trajectories. Three markers are installed on the chest and abdomen of each patient, and the trajectory data of the markers moving along with the respiratory movement were recorded. An optical tracking sampling instrument with a sampling frequency of 26 Hz is used for sampling work.

### 3.2 Research Methods

**Figure 1:**
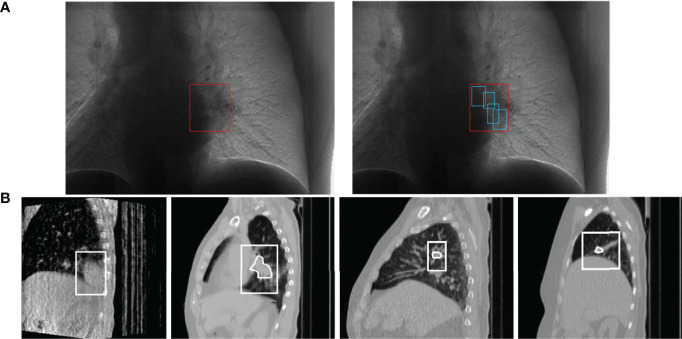
Schematic diagram of lung tumor motion tracking, **(A)** is the process of tumor localization (25). Each of **(B)** is a 4DCBCT (four-dimensional cone beam CT) sequence image of tumor tracking at different stages in a respiratory process, obtained by the EELKTA Synergy XVI system in the University of Tokyo Hospital (26).

In this study, we built a respiratory motion prediction model and used *in vitro* marker signals to predict tumor motion trajectories. **Figure 1** shows the process of tumor motion and machine positioning during radiotherapy. First, a tumor motion area in the lungs that follows the patient’s breathing is determined, and then, the tumor motion trajectory is further captured in this area. Considering the problem of mechanical and computer delays, the respiratory motion prediction model needs to determine the trajectory of the tumor after a period of delay, and finally perform radiotherapy to kill tumor cells.

**Figure 2:**
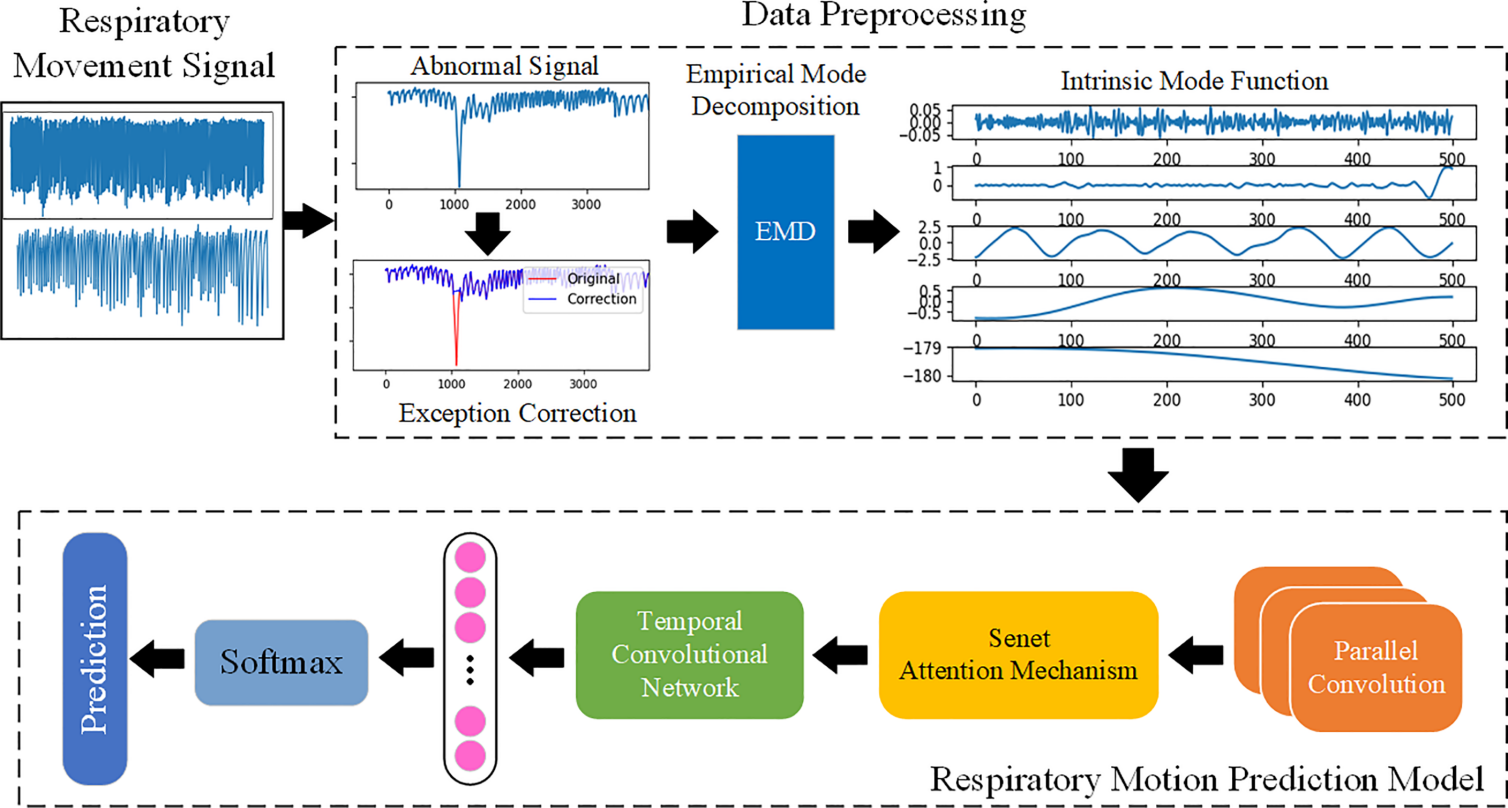
The overall framework of respiratory motion. Use EMD to fully extract the features of the respiratory motion signal and learn the features by building a prediction model based on deep learning, so as to achieve accurate prediction.

The overall framework of the breathing motion prediction method based on the deep CNN is shown in **Figure 2**, which is mainly divided into two steps (1): data preprocessing and feature extraction: abnormal detection and correction of respiratory signals and extraction of features using EMD decomposition signals (2); respiratory motion prediction model: a deep respiratory motion prediction model composed of multi-scale convolution neural network including SEnet attention mechanism and TCN for the prediction of respiratory position at different delay times from 200 to 500 ms.

#### 3.2.1 Data Preprocessing

In order to extract more information features and reduce the influence of interference information on prediction. First, the integrated model Bagging is used to detect and correct the abnormal interval, and then, the original series is decomposed into several IMFs containing different time scales by EMD algorithm, and finally, the dataset is divided as the input of depth prediction model.

##### 3.2.1.1 Remove Outliers

**Figure 3:**
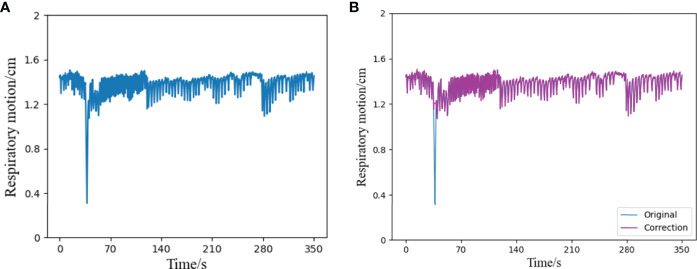
Schematic diagram of outlier correction and comparison, **(A)** is a segment of the original respiratory signal, which contains an abnormal state in a certain time interval, **(B)** shows the result of the respiratory signal after the outlier correction algorithm. Compared with the original signal, it can be seen that the part containing outliers has been successfully corrected, and the rest remain unchanged.

Because of the long time of data acquisition, tumor patients sometimes have actions such as coughing, sneezing, or speaking during the acquisition process, which will greatly interfere the stability of respiratory trajectory, resulting in relatively intensive abnormal signals of respiratory data at a certain time segment. Therefore, this paper uses Bagging to deal with abnormal signals. Bagging mainly samples T sampling sets containing m training samples, then trains a base learner on the basis of each sampling set, and finally combines these base learners together (27). **Figure 3** shows a comparison diagram before and after processing an abnormal signal.

##### 3.2.1.2 Empirical Mode Decomposition

Complex time series data will reduce the prediction precision of the prediction model, which will be alleviated to some extent by and the introduction of some decomposition algorithms in the pre-phase of data procession. Because the respiratory motion signal is a complex time series with non-linear, non-stationary, and univariate characteristics, when fitting this type of sequence with deep learning network, there are often problems such as gradient disappearance or explosion, and it is impossible to accurately identify the slight change characteristics of a certain time scale (28). Considering the multi-scale characteristics of time series, Fourier spectrum analysis and wavelet analysis are usually used to decompose the data to predict the better learning characteristics of the model. However, the limitations of these methods limit the operation of the prediction model to a certain extent, and empirical mode decomposition (EMD) can adaptively decompose complex signals. Compared with the above methods, EMD can more accurately reflect the original physical characteristics and local performance.

EMD decomposition is based on the following assumptions (29): the data have at least two extreme values (maximum and minimum); the local time–domain characteristics of the data are uniquely determined by the time scale between extreme points; if the signal is not extreme but contains an inflection point, then it can be differentiated once or more to obtain the extreme value. As for the given raw signal, *x*(*t*) (*t* = 1,2…*n*),the EMD algorithm decomposition is described as follows:

- Extraction of the maximum and minimum values of *x*(*t*): the upper and lower envelopes *X*_max_(*t*),*X*_max_(*t*) are formed by using the cubic spline difference to calculate their mean values *m*_1_:
- Extraction details:

(1) $$h_{t}\;=\;m(t)-m_{1}$$

- Judgment of whether *h_t_* IMF formation conditions: If it meets, then an IMF will be derived and the remaining volume *r*(*t*) = *x*(*t*)-*h*(*t*) will be in lieu of (*t*) ; if not, then *h_t_* will be in lieu of *x*(*t*).
- Repetition of the above steps: When the standard deviation (0.2-0.3) is met the iteration will be ended.
- After the decomposition process, can be replaced by the following formula:

(2) $$x\left(t\right)=\;\sum_{j=1}^{n}h_{j}(t)+r_{n}(t)$$

In this formula, *n* is the number of IMF; *h_j_* (*t*) (*j* = 1.2,…*n*) are IMFs; and *r*_n_(*t*) is the final residual error, which indicates the central trend of *x*(*t*).

**Figure 4:**
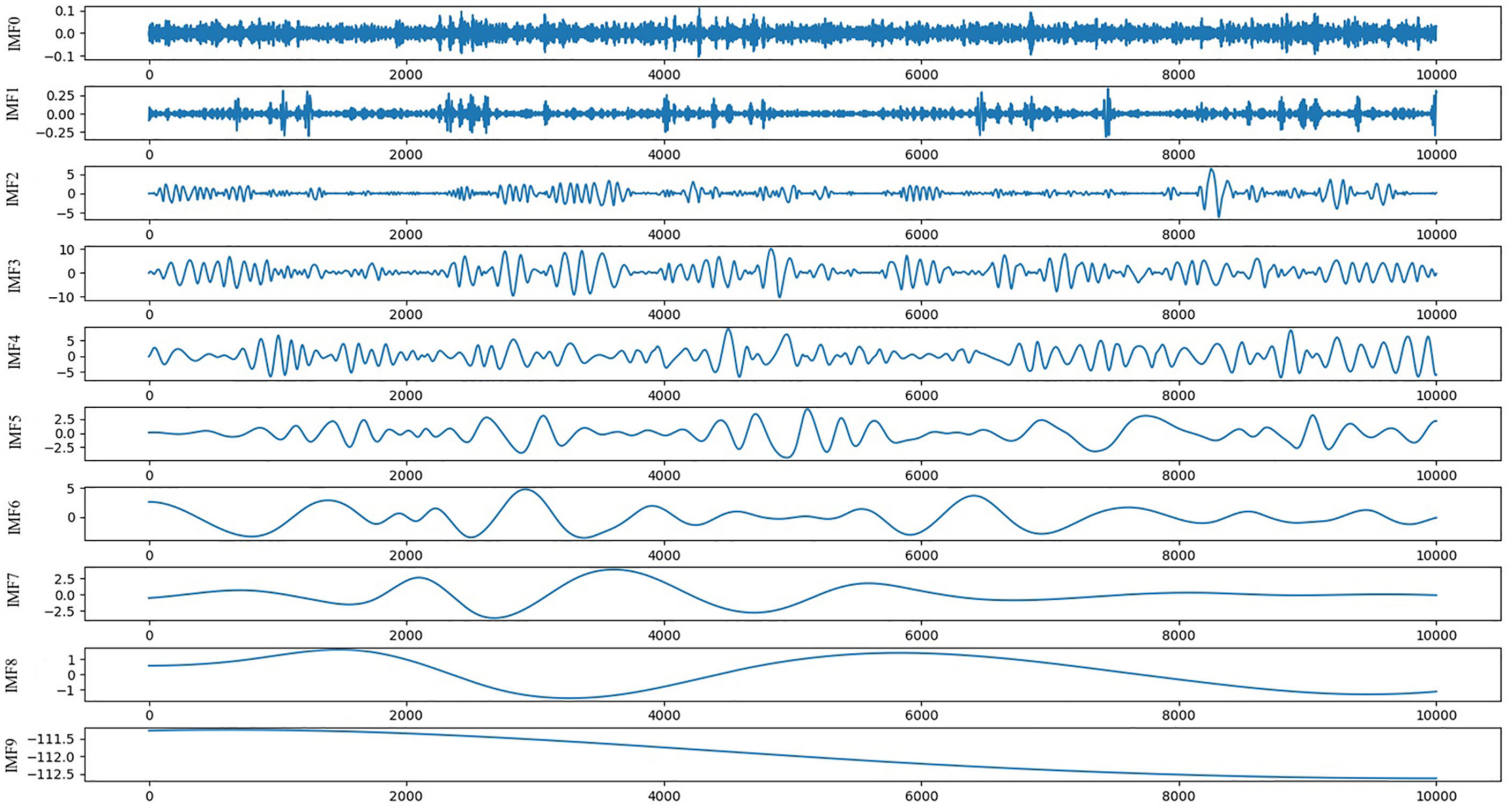
Schematic diagram of IMFs and Res decomposed by EMD.

For the generalization ability of the model, this paper uses the clinical respiratory data of 103 patients in the database and randomly selects a continuous signal (the total length of each signal is 10,000, about 7 min) from 306 respiratory trajectories as the model sample set. As shown in **Figure 4**, a series of length 10,000 is decomposed into nine IMF components and one residual (Res), and the physical meaning of each component of the IMF, whose order is divided according to the frequency from high to low, represents each frequency component of the raw signal. Because of the large amount of noise at high frequencies, the first two high-frequency IMF componets (*IMF*_0_,*IMF*_1_) are removed, and the remaining components will input the physical characteristics of the raw signal and into the prediction model. Because EMD is an adaptive decomposition, the respiratory data series of different patients will be decomposed into different amounts of IMF. Before being input into the network, it is necessary to supplement the number of IMFs of each original series in the whole database. The supplemented IMF components are filled with 0, so as to achieve a unified number of IMFs of each patient’s respiratory series.

##### 3.2.1.3 Division of Preprocessed Data

**Figure 5:**
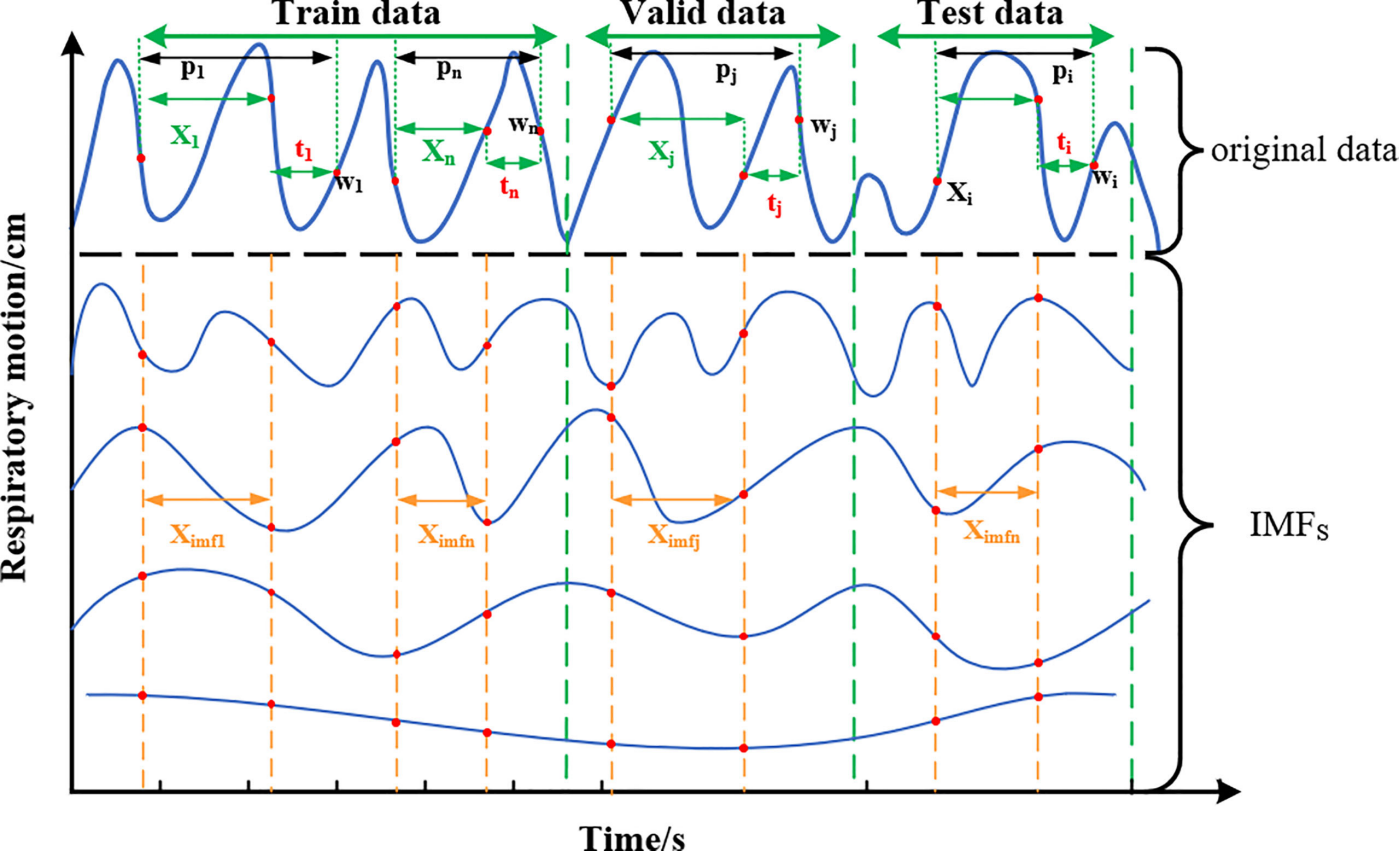
Schematic diagram of training set, verification set, and test set classification. The original data at each end are decomposed by EMD to form multiple IMFs, and the data of equal length are intercepted as the feature input.

The training set, validation set, and test set are partitioned among the filtered IMF components. As shown in the division diagram **Figure 5**, the original sequence P = (*p*_1_,*p*_2_,…,*p*_i_,…,*p_i_*_+_*_n_*) is divided in a ratio of 6:2:2. In addition, the training set is indicated as *P_train_* , *P_train_* = [*p*_1_,*p*_2_,…,*p_n_*]^T^ ; the valiadation set is denoted as *P_valid_* , *P_valid_* = [*p_j_*,…, *p_j+n_*]^T^; and the test set is denoted as *P_test_* , *P_test_* = [*p_j_*,…, *p_j+n_*]^T^. Take *P_train_* as an example, form *p*-1 top*_n_* all sequences are isometric sequences, and each sequence contains the original sequence (*X*_1_,*X*-2,…,*X_n_*) and the delay time of the predicted value (*t*_1_,…,*t_n_*).

Network model input: After decomposition of the original sequence *X*, IMFs correspond to part of *Ximfs*, *X_imfs_* = [*X_imf_*_1_, *X_imf_*_2_,…, *X_imf_*_1_, *X_imfi_*_+_*_n_*]^T^, which is a stationary sequence containing multidimensional features. Target prediction value (label): observation point (*w*_1_, *w_2_*,…,*w_n_*) after delay time *t* is the target prediction value, which is sampled from the original sequence and does not contain IMFs information. According to the equipment sampling frequency of 26 Hz, the corresponding delay time at *t_i_* = 3,5,10, and 13 is about 100, 200, 400, and 500 ms, respectively.

#### 3.2.2 Respiratory Motion Prediction Model

The deep convolution neural network model proposed in this paper for respiratory motion prediction includes three major parts. First, multi-scale convolution layers are used to extract features in parallel to find the optimal local sparse structure of the convolution network and obtain timing information fully. Second, the addition of a SEnet-based attention mechanism to the convolved feature channel increases the sensitivity of the model to the channel feature and automatically learns the importance of the different channel features. Last, TCNs are used to grasp long-time dependent information and assign each convolutional feature to a causal relationship, thereby predicting respiratory motion signals for a future period of time.

##### 3.2.2.1 Squeeze and Exception Module

CNN has the ability of characterization learning, translates invariant classification of input information according to the hierarchical structure, and fuses spatial and channel information in the local receiving domain of each layer of network to construct local features. A squeeze-and-excitation module is proposed on the basis of CNN by Hu et al. (30), which improves the CNN characterization ability by improving the spatial coding quality at the feature level and clearly establishing the interdependence between convolutional feature channels.

##### 3.2.2.2 Temporal Convolutional Network

The main characteristics of TCN include adopting a one-dimensional fully convolutional networks (FCNs) (31) to receive input sequences of any length as inputs and map them into output sequences of equal length at the same time; each time is calculated simultaneously, not serially on the time sequence, to improve the network operation efficiency; causal convolution is used, so that each convolution layer is causally related, which means that information “leakage” will not occur from future to the past. Briefly: TCN = 1D FCN + Causal convolutions (32).

###### 3.2.2.2.1 Causal Convolutions

**Figure 6:**
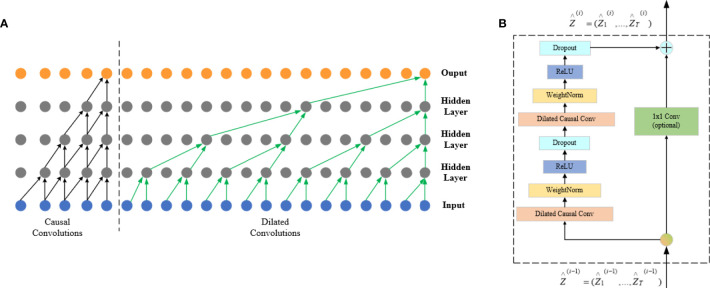
FCN architecture. The left half of **(A)** is a causal convolution schematic and the right half is a dilated convolution schematic, and **(B)** is the TCN residual block.

If the input sequence is shown as *X* = (*x*_1_.*x*_2_,…,*x_r_*), then the prediction *yt* of the moment *t* can only be obtained through *x*_1_ to *x_t_*_-1_, which is input before moment *t* as what has been shown in the left half of **Figure 6A**. If the filter is defined as *F* = (*f*_1_,*f*_2_,…,*f_k_*) and K is the number of filters, then the causal convolution at time *x_t_* is as follows:


(3) $$\left(F*X\right)\left(x_{t}\right)=\sum_{k=1}^{K}f_{k}x_{t-K+k}$$

There is a big defect in causal convolution. If a more distant *x_t_*_-_*_n_* is needed as input to enlarge the receptive field, then a large number of convolution layers are needed, which increases the network depth and easily causes problems such as gradient disappearance and poor fitting effect.

###### 3.2.2.2.2 Dilated Convolutions

Dilated convolution can be used to solve the above problems; meanwhile, it is also the convolution used by the TCN network. To obtain larger receptive field, the dilated convolution (*d*) introduces the concept of dilation factor, which allows the input interval adoption during the convolution. Adding to the dilation factor gives sequence X dilated convolution at *x_t_* at which the expansion factor is *d*:


(4) $$\left(F_{d}*X\right)\left(x_{t}\right)\;=\;\sum_{k=1}^{K}f_{k}\;x_{t-(K-k)d}$$

The right half of **Figure 6A** shows that *d* = 1 at input is a common convolution, with *d* = 2 for the first hidden layer and *d* = 4 for the second hidden layer, and the expansion factor increases exponentially by 2 as the network layer increases.

###### 3.2.2.2.3 Residual Connections

The residual connection is added to the TCN network, which allows the network to transmit information across layers and solves the problems of gradient disappearance or explosion of deep network, and learning the overall transformation of input *X* changes into learning the partial modification of input *X*. In the TCN, residual blocks are used to replace convolution layers, which include dilated convolution with two layers and non-linear mapping. In addition, a WeightNorm and Droput regularization network is used in each layer, with a linear rectification function (Relu) as the activation function as shown in **Figure 6B**.

##### 3.2.2.3 Network Layer of Respiratory Motion Prediction Model

**Figure 7:**
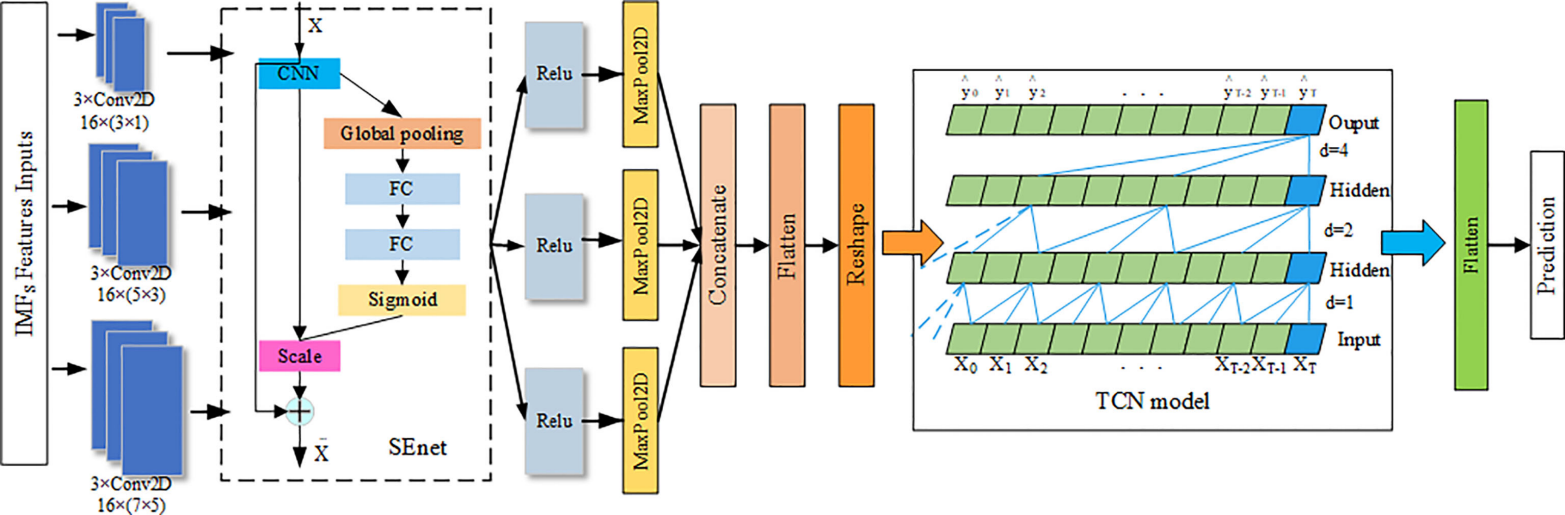
Network layer of respiratory prediction model.

The main body of respiratory motion prediction model is composed of multi-scale enhanced CNNs layer (CNN_SEnet) and a TCN layer. As shown in **Figure 7**, first, a multi-scale convolution channel is composed of a convolution layer containing different convolution kernels, and the sizes of each convolution kernel in each channel are 3 × 1, 5 × 3, and 7 × 5, respectively, with a step size of 1 and a convolution filter of 16. Setting convolution kernels at different scales allows the model to learn different local features in the sequence. For example, smaller convolution kernels can extract local subtle features and are more sensitive to instantaneous changes in the sequence; larger convolution kernels mainly extract local trend features and can control the overall features at a certain time scale. The input of the prediction model is *X_imfs_* = [*X_imf_*_1_, *X_imf_*_2_,…, *X_imf_*_1_, *X_imfi_*_+_*_n_*]^T^, in which the length of *X_imfn_* is the IMFs containing a certain time length, about 100 to 400, and the width is the IMFs with different frequency components formed by the original sequence after EMD decomposition, about 10 to 15. Its length–width ratio gap is so large that the convolution kernel size is no longer set as the conventional 3 × 3 or 5 × 5 but set the convolution kernel of the above size, which can highlight the time–domain characteristics when the frequency–domain characteristics are ensured. Each scale channel contains a convolution layer of three above parameters for adequately extracting feature information in the sequence.

Second, to enhance the information representation ability of CNNs layer, SEnet attention mechanism is added after each CNNs channel, and the weight coefficient of each channel after convolution is learned, so that the model has more discrimination ability for the characteristics of each channel. Its network parameters are detailed in the literature. The activation function Relu and the maximum pooling layer with a 2 × 2 window are then performed for extracting important features and discard irrelevant features.

Then, the output of the three scale channels is combined through the connecting layer to form a richer information feature. Afterward, the causal relationship of each feature can be found out through the TCN layer, and the future information is predicted through the historical information feature. The number of filters in this module is set as 32; the convolution kernel size is 3; the dilation factor grows by 2*^n^*; the number of stacks of residual blocks is 1, and the activation function is Relu. Last, the predicted target values were obtained through Flatten layer and full junctional layer.

#### 3.2.3 Evaluation Criteria

In this paper, the mean absolute error (MAE), RMSE, and R2 determination coefficient (R2_score) are used as evaluation indexes of respiratory prediction algorithm. MAE is the mean of the absolute value of the deviation between all individual observed value and the arithmetic mean. It is defined as follows:


(5) $$MAE\;=\;\frac{1}{N}\sum_{i=1}^{N}\left|y_{i}-y_{i}^{*}\right|$$

The RMSE is the square root of the ratio of the square of the deviation of the predicted value from the true value to the number of observations *n*, and it is defined as follows:


(6) $$RMSE\;=\;\sqrt{\frac{1}{N}\sum_{i=1}^{N}{\left(y_{i}-y_{i}^{*}\right)}^{2}}$$

R2_score is the overall fit of the regression equation, and the closer the value of R2 is to 1, the better the fit of the regression equation to the observed value is, which can be defined as follows:


(7) $$RMSE\;=\;1-\frac{\sum_{i}{\left(y_{i}-y_{i}^{*}\right)}^{2}}{\sum_{i}{\left({\bar{y}}_{i}-y_{i}^{*}\right)}^{2}}$$

In this equation, *N* is the number of data points; *y* is the actual respiratory motion trace; y* is the trajectories of respiratory motion prediction; and $\sum_{i}{\left({\bar{y}}_{i}-y_{i}^{*}\right)}^{2}$ is a benchmark model in the field of machine learning.

## 4 Results And Discussion

### 4.1 Results

**Figure 8:**
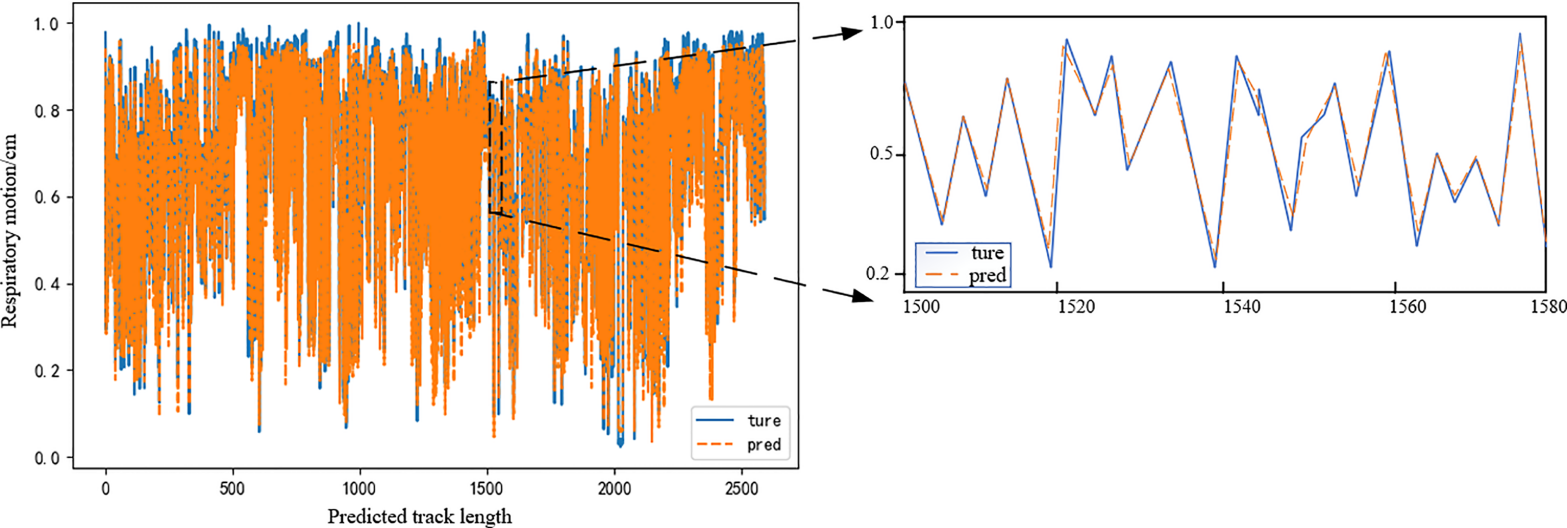
Actual breathing trajectory and predicted trajectory. Model delay time is 400 ms.

**Table 1** and **Figure 8**, respectively, show the experimental results of the proposed EMD-SEnet-TCN multi-channel depth prediction model in this paper; in addition, the prediction results in this paper are all calculated according to the following parameters: epochs = 100, batch size = 128, optimizer = Adam, and learning rate = 0.001. Judging from the results, although the prediction precision decreases with the increase of delay time (*t_i_*), the prediction accuracy is still ensured to some extent; when the length of model input data is increased, the network does not present gradient explosion or disappearance problems, which indicates that the proposed algorithm in this paper has the ability to overcome long-distance dependence and can make full use of historical information to predict the future information.

**Table 1:** Results of respiratory prediction algorithm.

Input Length (Xi)	Latency (ms)	MAE (mm)	RMSE (mm)	R2 (None)
50	120 (*t_i_* = 3)	0.009022	0.022503	0.989431
100	200 (*t_i_* = 5)	0.016584	0.031588	0.979483
200	400 (*t_i_* = 10)	0.035926	0.053782	0.941398
400	500 (*t_i_* = 13)	0.048367	0.068925	0.908258

**Figure 9:**
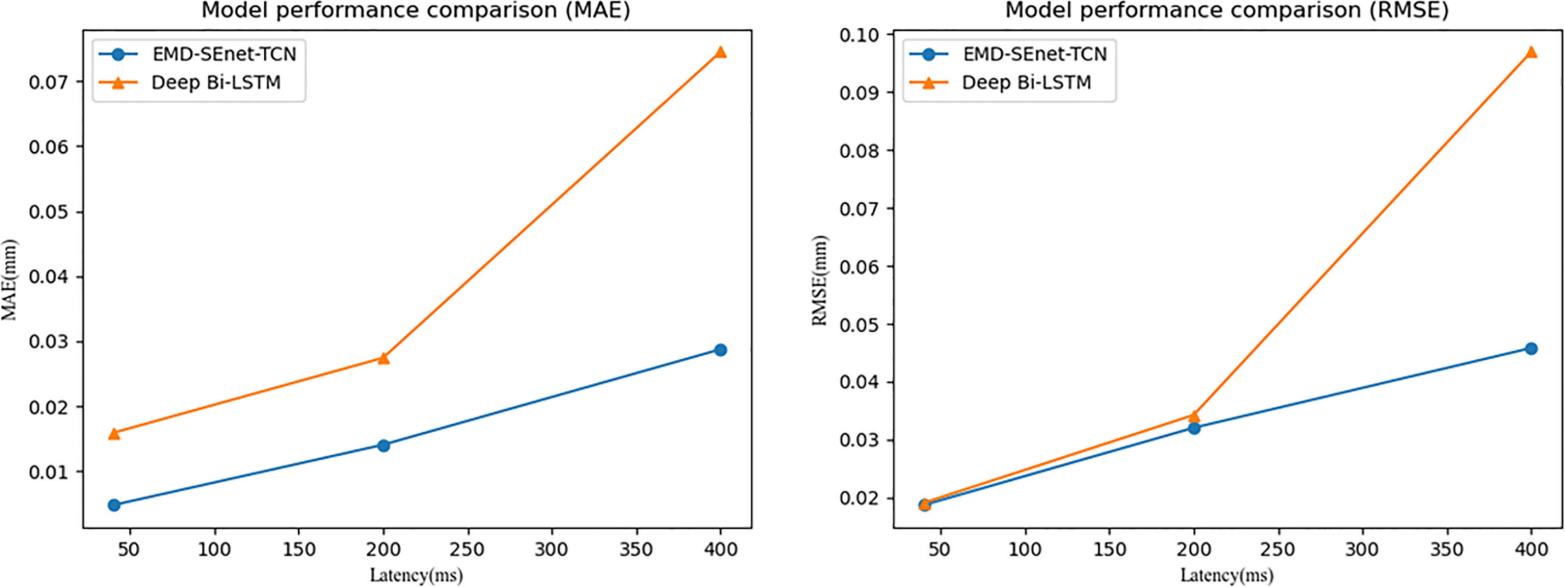
Model performance comparison.

To verify the higher prediction precision of the model in this paper, a comparison is made with the Deep BiLSTM model proposed by Wang et al. (20) with the same dataset. **Figure 9** shows the comparison of these two algorithmic models under the same parameters (*X_i_* = 50, *t_i_* = 1.5, and 10). It can be seen from the figure that the prediction precision of the algorithm proposed in this paper is better at different delay times under the MAE and RMSE evaluation indexes.

Because of the limitations of different input samples, preprocessing operations, and experimental platforms under different models, to illustrate the superiority of this model more clearly, a comparison among three depth prediction models is conducted, including multi-convolution combined with BiLSTM network (CNN-BiLSTM), multi-channel convolution combined with TCN model (CNN-TCN), and multi-channel convolution combined with BiLSTM based on EMD (EMD-CNN-BiLSTM). **Table 2** shows the performance comparison results of the proposed algorithm (EMD-SEnetTCN) with the above three models at *X_i_* = 100 and delay times at 80, 150, 240, 300, 400, 450, and 520 ms (*t_i_* = 2, 4, 6, 8, 10, 12, and 14).

**Table 2:** Results comparison of different respiratory prediction models.

Prediction model	Latency (ms)	MAE (mm)	RMSE (mm)	R2 (None)
EMD-SEnet-TCN	80	0.008797	0.01814	0.993157
	150	0.016442	0.026901	0.985422
	240	0.021495	0.033960	0.975841
	300	0.028391	0.042698	0.960636
	400	0.031789	0.0491499	0.951819
	450	0.038560	0.058295	0.928711
	520	0.045638	0.064746	0.910043
CNN-BiLSTM	80	0.013164	0.020244	0.994183
	150	0.015645	0.021739	0.989963
	240	0.026275	0.032612	0.966891
	300	0.040191	0.051979	0.939334
	400	0.044840	0.060412	0.917064
	450	0.051416	0.071154	0.890039
	520	0.059593	0.080446	0.857721
CNN-TCN	80	0.007193	0.009572	0.997983
	150	0.020939	0.028718	0.985953
	240	0.022003	0.031190	0.978862
	300	0.029881	0.045487	0.961351
	400	0.040356	0.054568	0.932332
	450	0.048732	0.070259	0.893678
	520	0.054356	0.077895	0.869860
EMD-CNN-BiLSTM	80	0.010331	0.022604	0.989376
	150	0.017215	0.025713	0.986681
	240	0.0257432	0.038429	0.969060
	300	0.029316	0.045936	0.954440
	400	0.037918	0.054366	0.937525
	450	0.040603	0.060994	0.911850
	520	0.048301	0.065581	0.907710

**Figure 10:**
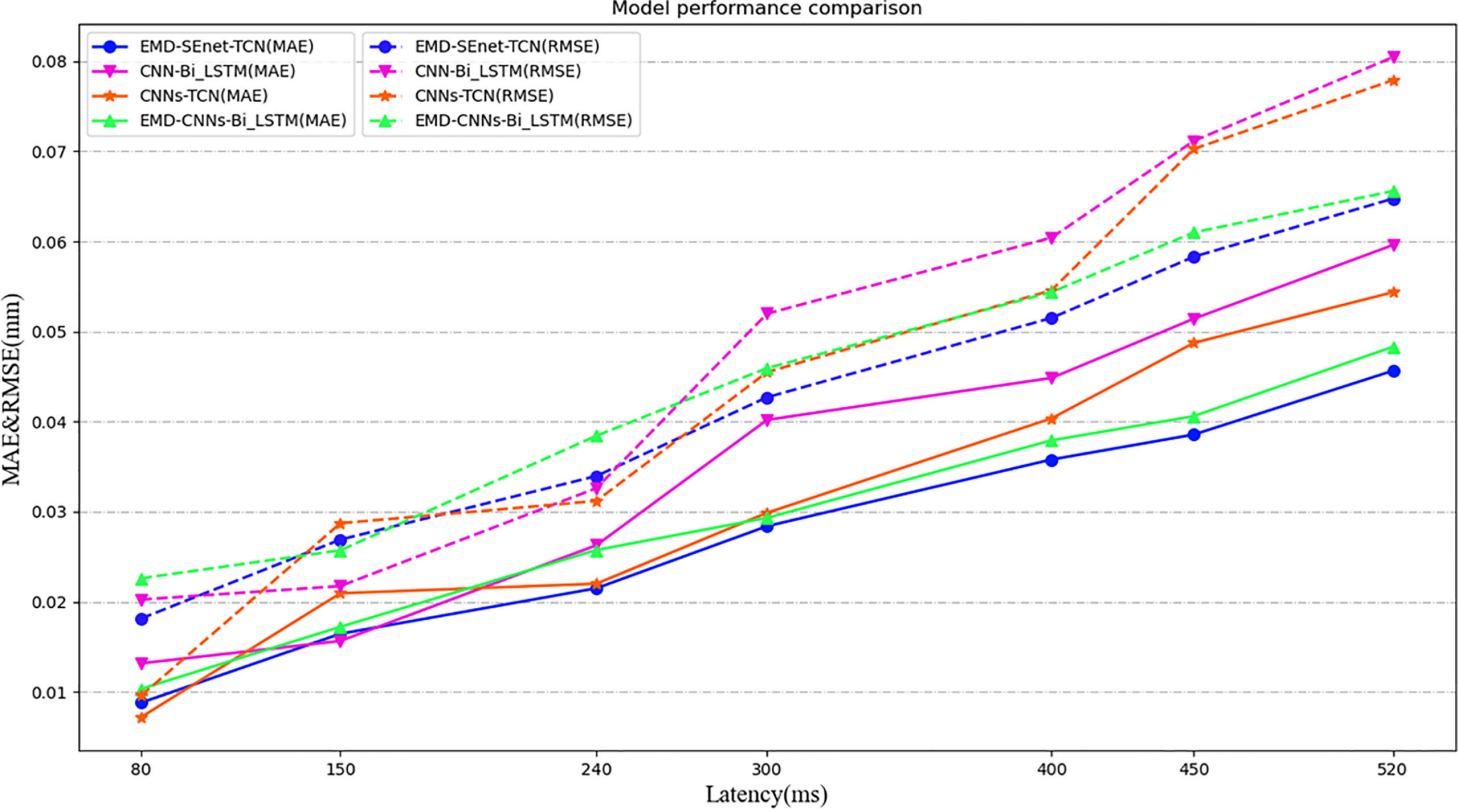
Model performance comparison under different depths and different evaluation criteria. Under different predictive evaluation indicators, the performance of this model is compared with the other three models. The blue represents the model of this paper, the solid line represents the MAE indicator, and the dashed line represents the RMSE indicator.

As shown in **Figure 10**, the prediction precision of each model is high, and there is no significant difference when the delay time is shorter than 240 ms. The MAE and RMSE are about 0.72% ~ 0.18% and 0.21% ~ 0.28%, respectively. When the delay time exceeds 240 ms, the better performance of EMD-SEnet-TCN becomes more and more obvious. To meet the clinical requirements, 400 ms is used as the standard delay time. Compared with CNN-TCN, the precision decrease of MAE and RMSE are by 13.7% and 9.2%, respectively, whereas for R2_score, the precision increases by 2%. The difference between these two models is whether EMD is used or not. Judging from the results, EMD is very effective for improving the precision of the model. Compared with CNN-BiLSTM, the precision values of MAE and RMSE decreased by 15% and 18.3%, respectively, whereas the precision value of R2_score increased by 1.4%; compared with EMD-CNN-BiLSTM, the precision values of MAE and RMSE decreased by 8.4% and 9.1%, respectively, whereas the precision value of R2_score increased by 1%, of MAE decreased by 8.4%, of RMSE decreased by 9.1%, and of R2_score increased by 1%. The prediction precision of this model is very close to that of this paper due to the similar structure of the two depth models and the difference lies in TCN and BiLSTM. EMD-SEnet_TCN not only has higher precision but also improves of prediction update time. The results show that, compared with other prediction models, the model in this paper has excellent performance at different delay times, and the prediction model performance will be further improved with the increase of delay time.

**Figure 11:**
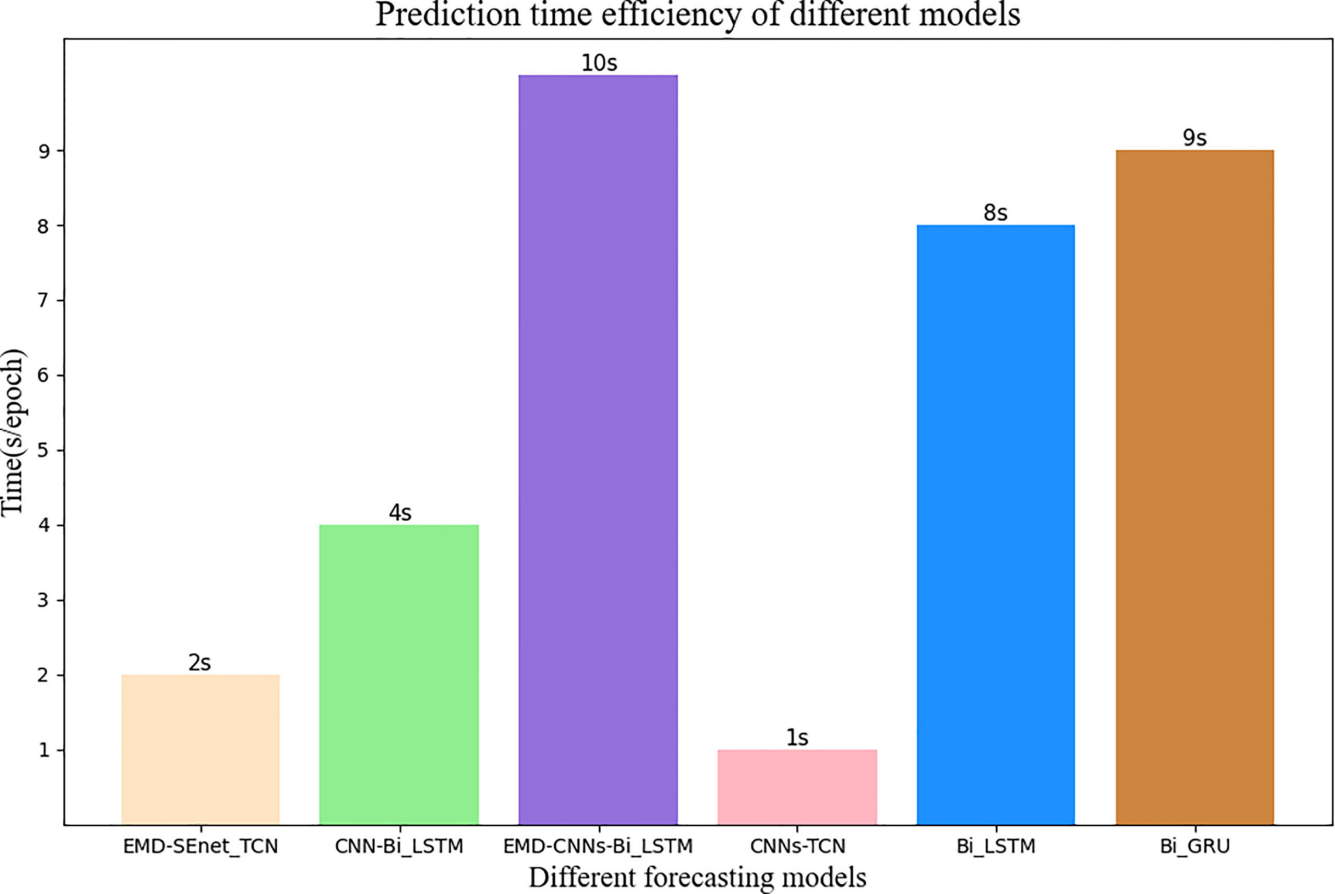
Average update time of different prediction models. The number at the top of the bar graph represents time, and the color of wheat represents the model of this article.

**Figure 11** shows the prediction update time of different depth models in seconds per epoch. Although EMD-CNN-BiLSTM is slightly inferior to the model proposed in this paper in terms of prediction precision, the update time has reached 10 s per epoch, which is much longer than the update time of EMD-SEnet_TCN (2 s per epoch), failing to meet the clinical requirements; whereas the update time of CNN-TCNs is the shortest, only 1 s per epoch, without meeting the standard of prediction pricision; as for other prediction models, all perform poorly in terms of precision or update time. In general, the prediction model proposed in this paper greatly reduces the average update time with the guarantee of high prediction precision, so that the network can predict the target value quickly and accurately.

**Figure 12:**
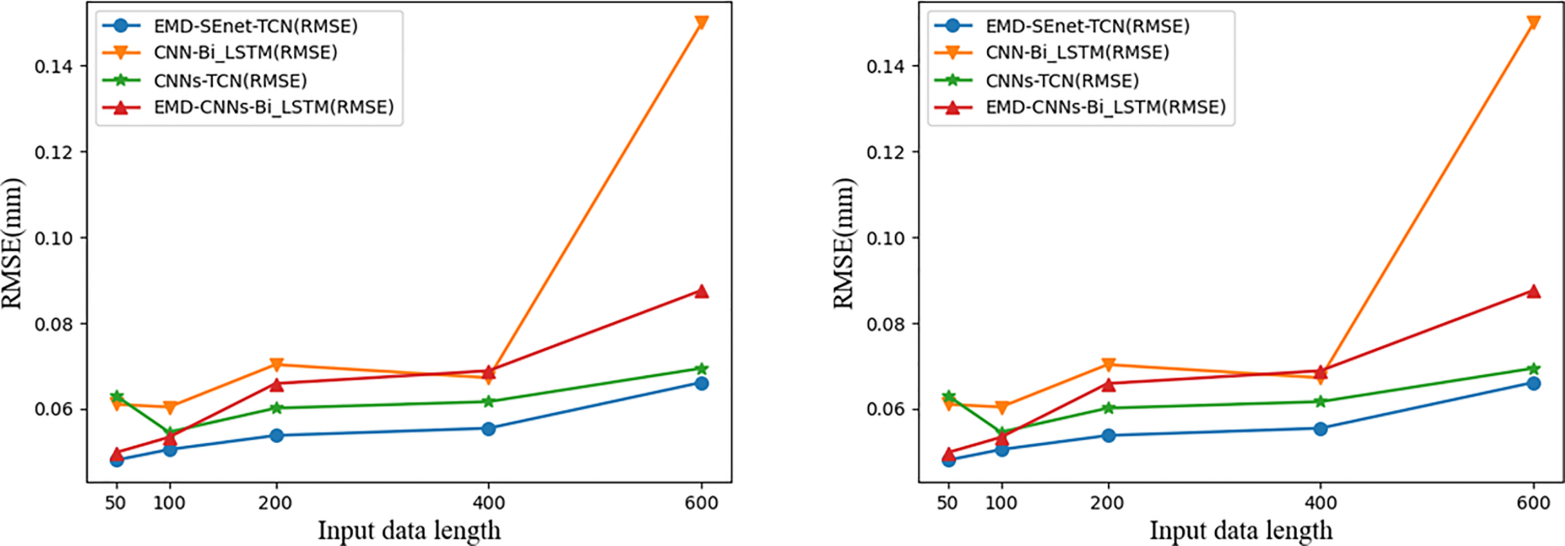
Comparison of prediction results of input data with different lengths. Blue represents the model of this article.

The input data length of the model affects the prediction precision to a certain extent. Generally, to lower the prediction error, the input data segment should be located near the target prediction value because the farther the distance is, the weaker the correlation is. In addition, if the data is too long, then there will be problems such as increased training time of the prediction model and gradient disappearance or explosion. To study the effect of different lengths of input data on the prediction results, the prediction errors of different data with lengths of 50, 100, 200, 400, and 600 at a delay time of 400 ms (*t_i_* = 10) are compared. The results are shown in **Figure 12**.

With the increase of input data (*X_i_*) the prediction errors of different models increase, among which the gradient of CNN-BiLSTM disappears at *X_i_* = 600 and both MAE and RMSE increase abnormally; EMD-CNN-BiLSTM and CNN-BiLSTM have better prediction precision when *X_i_* is small, but the prediction error increases rapidly when *X_i_* is big; CNN-TCN has a more stable prediction error fluctuation at different *X_i_* whereas that of MAE and RMSE are big; comparing the above three models, EMD-SEnet_TCN displays excellent prediction performance in that it can cope with sequence information of various lengths and ensure certain prediction precision.

### 4.2 Discussion

Choosing different optimizer (Op) and learning rate (Lr) will affect the prediction results of deep prediction model. The optimizer is used to update and calculate the network parameters affecting the training and output of model, so that they approximate or reach the optimal value to minimize (or maximize) the loss function. The learning rate determines whether the objective function can converge to the local minimum value and when it can converges to the minimum value. The appropriate learning rate can make the target function converge to the local minimum value at appropriate time. SGD is a relatively commonly used optimizer, in which noise will be added when the gradient is randomly selected, and the update weight value does not reach the global optimum, which makes the accuracy rate decrease; Adagrad adopts an adaptive learning rate optimization algorithm to update the low-frequency parameters greatly while update the high-frequency parameters less; Adadelta is an improvement of Adagrad because it has an exponential decay average; RMSprop changes the gradient accumulation of Adagrad into an exponentially weighted moving average, improving the effect under non-convex settings; Adam combines the momentum advantages of RMSprop with SGD to form an optimizer with better performance.

Different optimizers display differently in various tasks, and it is not necessarily that the more advanced the version is, the better its results are. To select a better optimizer, the comparison of different optimizers is performed in **Table 3**. The learning rate controls the update speed of model parameters–Lr is too small, it will greatly reduce the network convergence rate and increase the training time; if it is too large, then it will lead to parameters oscillating on both sides of the optimal solution. **Table 3** below shows the prediction model performance results of different sizes of learning rates (0.1, 0.01, 0.001, and 0.0001).

**Table 3:** Effect of different parameters (Op, Lr) on EMD-SEnet_TCN.

Parameters	MAE (mm)	RMSE (mm)	R2 (None)
Op = SGD	0.087130	0.116361	0.702972
Op = Adam	0.035789	0.051499	0.941819
Op = Adagrad	0.049455	0.069139	0.895137
Op = Adadelta	0.166532	0.200430	0.118740
Op = RMSprop	0.039013	0.060148	0.910473
Lr = 0.1	0.308492	0.375175	−2.08768
Lr = 0.01	0.037244	0.053731	0.936662
Lr = 0.001	0.034789	0.049149	0.951819
Lr = 0.0001	0.041593	0.057317	0.927934

All the results in **Table 3** are based on EMD-Senet-TCN prediction model with epochs = 100, batch size = 128, *X_i_* = 100, *t_i_* = 10 (400 ms). From **Table 3**, it can be seen that Op uses Adam. MAE and RMSE are the smallest and their prediction is the most accurate. Although Adadelta is an advanced version of Adagrad, it is not very effective when applied under the prediction model in this paper. The different learning rate settings were all obtained under Op = Adam, and the best result was obtained when *Lr* = 0.001, where when *Lr* = 0.01, the learning rate is too large to result in a model that could not converge and the regression coefficient was negative. It can be seen that the model in this paper uses Op = Adam and *Lr* = 0.001 to the best prediction results.

## 5 Conclusion

Respiratory motion brings great difficulties to the treatment of thoracoabdominal tumors, and respiratory motion prediction models are extremely important for precision radiotherapy. In this paper, a depth prediction model (EMD-SEnet-TCN) is proposed for the application of respiratory motion signals in radiation therapy for patients with cancer. The method was validated by using respiratory motion signals from multiple patients with malignant tumors in the database of the Institute of Robotics and Cognitive Systems, University of Lübeck, Germany. The results of this paper show that (1) the depth prediction model method proposed in this paper is superior to other benchmark models in terms of delay prediction precision and time update efficiency (2); it verifies that the decomposition of complex respiratory motion signals by using EMD can further improve the prediction precision of the prediction model (3); the multi-scale CNN containing attention mechanisms has a better feature extraction ability for finite IMFs of respiratory motion signals. This work solves one of the major challenges for precise prediction of the state of patient respiratory motion signals, and in medical practice, the proposed method has important practical significance for precision radiation therapy.

The present study has some limitations. The first one is the correlation between the external respiratory signal and the internal tumor motion. In order for our technique to be applied clinically, another model needs to be designed to realize the correlation analysis in the future. The second is that whether the prediction technology in this paper achieves clinical application is the key to future research. On the basis of complying with legal and ethical requirements and respecting patient privacy, it is very important to determine a medical analysis platform that applies the deep learning framework in the future.

## Data Availability Statement

Publicly available datasets were analyzed in this study. This data can be found at http://signals.rob.uni-luebeck.de/.

## Author Contributions

Conceptualization: LS and JZ; Formal analysis: LS and SH; Supervision: ZK and WJ; Investigation: YC and ZZ; Writing—original draft: JZ and SH; Writing—review and editing: LS and JZ. All authors contributed to the article and approved the submitted version.

## Funding

Funding sources: the Jilin Provincial Department of Science and Technology (Grant/Award Number: No.20190201195JC.20200601004JC.20200301054RQ.20200404207YY), Science and Technology Development Plan of Jilin Province (Grant/Award Number: 20200403120SF), Natural Science Foundation of Jilin Province (Grant/Award Number: 20210101477JC), and the National Natural Science Foundation of China for supporting the research in this article (Grant/Award Number: No.61502052).

## Conflict of Interest

The authors declare that the research was conducted in the absence of any commercial or financial relationships that could be construed as a potential conflict of interest.

## Publisher’s Note

All claims expressed in this article are solely those of the authors and do not necessarily represent those of their affiliated organizations, or those of the publisher, the editors and the reviewers. Any product that may be evaluated in this article, or claim that may be made by its manufacturer, is not guaranteed or endorsed by the publisher.

## References

[1] Zhao B Yang Y Li T Li X Heron DE Huq MS . Dosimetric Effect of Intrafraction Tumor Motion in Phase Gated Lung Stereotactic Body Radiotherapy. Med Phys (2012) 39:6629–37. doi: 10.1118/1.4757916 23127057

[2] Shirato H Suzuki K Sharp GC Fujita K Onimaru R Fujino M . Speed and Amplitude of Lung Tumor Motion Precisely Detected in Four-Dimensional Setup and in Real-Time Tumor-Tracking Radiotherapy. Int J Radiat Oncol Biol Phys (2006) 64:1229–36. doi: 10.1016/j.ijrobp.2005.11.016 16504762

[3] Rydhög JS de Blanck SR Josipovic M Jølck RI Larsen KR Clementsen P . Target Position Uncertainty During Visually Guided Deep-Inspiration Breath-Hold Radiotherapy in Locally Advanced Lung Cancer. Radiother Oncol (2017) 123:78–84. doi: 10.1016/j.radonc.2017.02.003 28245908

[4] Herfarth K Debus J Lohr F Bahner M Fritz P Höss A . Extracranial Stereotactic Radiation Therapy: Set-Up Accuracy of Patients Treated for Liver Metastases. Int J Radiat Oncol Biol Phys (2000) 46:329–35. doi: 10.1016/S0360-3016(99)00413-7 10661339

[5] Oh SA Yea JW Kim SK . Statistical Determination of the Gating Windows for Respiratory-Gated Radiotherapy Using a Visible Guiding System. PloS One (2016) 11:e0156357. doi: 10.1371/journal.pone.0156357 27228097 PMC4881953

[6] Murphy MJ . Tracking Moving Organs in Real Time. Semin Radiat Oncol (Elsevier) (2004) 14:91–100. doi: 10.1053/j.semradonc.2003.10.005 14752737

[7] Vedam S Kini V Keall P Ramakrishnan V Mostafavi H Mohan R . Quantifying the Predictability of Diaphragm Motion During Respiration With a Noninvasive External Marker. Med Phys (2003) 30:505–13. doi: 10.1118/1.1558675 12722802

[8] Ozhasoglu C Murphy MJ . Issues in Respiratory Motion Compensation During External-Beam Radiotherapy. Int J Radiat Oncol Biol Phys (2002) 52:1389–99. doi: 10.1016/S0360-3016(01)02789-4 11955754

[9] Fayad H Pan T François Clement J Visvikis D . Correlation of Respiratory Motion Between External Patient Surface and Internal Anatomical Landmarks. Med Phys (2011) 38:3157–64. doi: 10.1118/1.3589131 PMC3379968 21815390

[10] Richter L Ernst F Martens V Matthäus L Schweikard A . Client/server Framework for Robot Control in Medical Assistance Systems. Int J Comput Assist Radiol Surg (2010) 5:306–7.

[11] Depuydt T Verellen D Haas O Gevaert T Linthout N Duchateau M . Geometric Accuracy of a Novel Gimbals Based Radiation Therapy Tumor Tracking System. Radiother Oncol (2011) 98:365–72. doi: 10.1016/j.radonc.2011.01.015 21316786

[12] Smith RL Abd Rahni AA Jones J . A Kalman-Based Approach With Em Optimization for Respiratory Motion Modeling in Medical Imaging. IEEE Trans Radiat Plasma Med Sci (2018) 3(4):410–20. doi: 10.1109/TRPMS.2018.2879441

[13] Hong S Bukhari W . Real-Time Prediction of Respiratory Motion Using a Cascade Structure of an Extended Kalman Filter and Support Vector Regression. Phys Med Biol (2014) 59:3555. doi: 10.1088/0031-9155/59/13/3555 24909152

[14] Ernst F Schlaefer A Schweikard A . Prediction of Respiratory Motion With Wavelet-Based Multiscale Autoregression. Int Conf Med Imag Comput Computer-Assisted Intervent (2007) 4792:668–75. doi: 10.1007/978-3-540-75759-7_81 18044626

[15] Ernst F Schweikard A . Prediction of Respiratory Motion Using a Modified Recursive Least Squares Algorithm. CURAC (2008) 8:157–60. doi: 10.1.1.149.4134

[16] Homma N Sakai M Takai Y . Time Series Prediction of Respiratory Motion for Lung Tumor Tracking Radiation Therapy. In: Proceedings of the 10th WSEAS International Conference on Neural Networks. Prague, Czech Republic: World Scientific and Engineering Academy and Society (WSEAS) (2009), 126–31. doi: 10.5555/1561799.1561822

[17] Tsai TI Li DC . Approximate Modeling for High Order Non-Linear Functions Using Small Sample Sets. Expert Syst Appl (2008) 34:564–9. doi: 10.1016/j.eswa.2006.09.023

[18] Sun W Jiang M Ren L Dang J You T Yin F . Respiratory Signal Prediction Based on Adaptive Boosting and Multi-Layer Perceptron Neural Network. Phys Med Biol (2017) 62:6822. doi: 10.1088/1361-6560/aa7cd4 28665297 PMC5555420

[19] Wei W Ke Q Nowak J Korytkowski M Scherer R Woźniak M . Accurate and Fast Url Phishing Detector: A Convolutional Neural Network Approach. Comput Networks (2020) 178:107275. doi: 10.1016/j.comnet.2020.107275

[20] Wang R Liang X Zhu X Xie Y . A Feasibility of Respiration Prediction Based on Deep Bi-Lstm for Real-Time Tumor Tracking. IEEE Access (2018) 6:51262–8. doi: 10.1109/ACCESS.2018.2869780

[21] Yu S Wang J Liu J Sun R Kuang S Sun L . Rapid Prediction of Respiratory Motion Based on Bidirectional Gated Recurrent Unit Network. IEEE Access (2020) 8:49424–35. doi: 10.1109/ACCESS.2020.2980002

[22] Tang S Andres B Andriluka M Schiele M . Multi-person Tracking by Multicut and Deep Matching. Computer Vision - {ECCV} 2016 Workshops - Amsterdam, The Netherlands, October 8-10 and 15-16, 2016, Proceedings, Part {II} (2016) 9914:100–11. doi: 10.1007/978-3-319-48881-3\_8

[23] Teo TP Ahmed SB Kawalec P Alayoubi N Bruce N Lyn E . Feasibility of Predicting Tumor Motion Using Online Data Acquired During Treatment and a Generalized Neural Network Optimized With Offline Patient Tumor Trajectories. Med Phys (2018) 45:830–45. doi: 10.1002/mp.12731 29244902

[24] Ernst F . Compensating for Quasi-Periodic Motion in Robotic Radiosurgery. Berlin, Germany: Springer Science & Business Media (2011).

[25] Shumeng H Shanda M Wei W Dongshan F . Lung Tumor Motion Tracking Method and Clinical Evaluation Based on Dual-Energy X-Ray Fluoroscopic Imaging. J Tianjin Med Univ (2020) 26:6. doi: 10.1109/TKDE.2016.2609424

[26] Pohl M Uesaka M Demachi K Chhatkuli RB . Prediction of the Motion of Chest Internal Points Using a Recurrent Neural Network Trained With Real-Time Recurrent Learning for Latency Compensation in Lung Cancer Radiotherapy. Computer Med Imaging Graphics (2021) 91:101941. doi: 10.1016/j.compmedimag.2021.101941 34265553

[27] Oza NC Russell SJ . 2005 IEEE International Conference on Systems, Man and Cybernetics. Online Bagging and Boosting (2001) 3:2340–5. doi: 10.1109/ICSMC.2005.1571498

[28] Perais A Seznec A . Eole: Combining Static and Dynamic Scheduling Through Value Prediction to Reduce Complexity and Increase Performance. ACM Trans Comput Syst (TOCS) (2016) 34:1–33. doi: 10.1145/2870632

[29] Huang NE Shen Z Long SR Wu MC Shih HH Zheng Q . The Empirical Mode Decomposition and the Hilbert Spectrum for Nonlinear and non-Stationary Time Series Analysis. Proc R Soc London. Ser A: Mathe Phys Eng Sci (1998) 454:903–95. doi: 10.1098/rspa.1998.0193

[30] Hu J Shen L Sun G . Squeeze-And-Excitation Networks. In: 2018 {IEEE} Conference on Computer Vision and Pattern Recognition, (CVPR) 2018, Salt Lake City, UT, USA, June 18-22, 2018. Computer Vision Foundation / (IEEE) Computer Society (2018) 7132–41. doi: 10.1109/CVPR.2018.00745

[31] Long J Shelhamer E Darrell T . Fully Convolutional Networks for Semantic Segmentation. In: (IEEE) Conference on Computer Vision and Pattern Recognition, {CVPR} 2015, Boston, MA, USA, June 7-12, 2015. (IEEE) Computer Society (2015) 3431–40. doi: 10.1109/CVPR.2015.7298965

[32] Bai S Kolter JZ Koltun V . An Empirical Evaluation of Generic Convolutional and Recurrent Networks for Sequence Modeling. arXiv (2018) abs/1803.01271. doi: 10.48550/arXiv.1803.01271

